# An epigenetic biomarker combination of PCDH17 and POU4F2 detects bladder cancer accurately by methylation analyses of urine sediment DNA in Han Chinese

**DOI:** 10.18632/oncotarget.6666

**Published:** 2015-12-19

**Authors:** Yongqiang Wang, Yuan Yu, Rui Ye, Duo Zhang, Qiaoling Li, Dan An, Lu Fang, Youcheng Lin, Yong Hou, Abai Xu, Yu Fu, Wei Lu, Xin Chen, Mingwei Chen, Meng Zhang, Huiling Jiang, Chuanxia Zhang, Pei Dong, Chong Li, Jun Chen, Guosheng Yang, Chunxiao Liu, Zhiming Cai, Fangjian Zhou, Song Wu

**Affiliations:** ^1^ Department of Urology, Sun Yat-sen University Cancer Center, Collaborative Innovation Center for Cancer Medicine, State Key Laboratory of Oncology in South China, Guangzhou, 510060, China; ^2^ The Affiliated Luohu Hospital of Shenzhen University, Shenzhen Luohu Hospital Group, Shenzhen 518000, China; ^3^ BGI-Shenzhen, Shenzhen 518083, China; ^4^ Anhui Medical University, Hefei 230032, China; ^5^ Department of Urology, Zhujiang Hospital of Southern Medical University, Guangzhou 510280, China; ^6^ The Second People's Hospital of Guangdong Province, Guangzhou 510310, China; ^7^ The First Affiliated Hospital, Sun Yat-Sen University, Guangzhou 510080, China; ^8^ Zhongshan School of Medicine, Sun Yat-Sen University, Guangzhou 510080, China; ^9^ CAS Key Laboratory of Infection and Immunity, Institute of Biophysics, Chinese Academy of Sciences, Beijing 100101, China; ^10^ The Third Affiliated Hospital, Sun Yat-Sen University, Guangzhou 510630, China; ^11^ Department of Urological Surgery, Shenzhen Second People's Hospital, The First Affiliated Hospital of Shenzhen University, Shenzhen 518000, China

**Keywords:** bladder cancer, epigenetics, methylation, biomarkers, qMSP

## Abstract

To develop a routine and effectual procedure of detecting bladder cancer (BlCa), an optimized combination of epigenetic biomarkers that work synergistically with high sensitivity and specificity is necessary. In this study, methylation levels of seven biomarkers (*EOMES*, *GDF15*, *NID2*, *PCDH17*, *POU4F2*, *TCF21*, and *ZNF154*) in 148 individuals—which including 58 urothelial cell carcinoma (UCC) patients, 20 infected urinary calculi (IUC) patients, 20 kidney cancer (KC) patients,20 prostate cancer (PC) patients, and 30 healthy volunteers (HV)—were quantified by qMSP using the urine sediment DNA. Receiver operating characteristic (ROC) curves were generated for each biomarker. The combining predictors of possible combinations were calculated through logistic regression model. Subsequently, ROC curves of the three best performing combinations were constructed. Then, we validated the three best performing combinations and *POU4F2* in another 72 UCC, 21 IUC, 26 KC and 22 PC, and 23 HV urine samples. The combination of *POU4F2/PCDH17* has yielded the highest sensitivity and specificity of 90.00% and 93.96% in all the 312 individuals, showing the capability of detecting BlCa effectively among pathologically varied sample groups.

## INTRODUCTION

Bladder cancer (BlCa), in the United States only, is currently accounted for the diagnosis of approximately 608,620 patients and consisted 4% of cancer survivors by the end of 2013, while an additional 74,690 cases are expected to be diagnosed in 2014 [1]. In China, BlCa is the seventh common cancer in men, but is not the top ten common cancer in women [2]. For all stages of BlCa combined, the 5-year relative survival rate is 77.9%. When BlCa is diagnosed in early stage (51% of cases), the 5-year survival rate is 96.4% [3]. In order to improve the prognosis and survival rate of BlCa patients, accurate early-stage diagnosis is essential. Traditionally, the diagnosis of BlCa that has been widely applied by cystoscopy. Even though cystoscopy is deemed to be the gold standard for diagnosing BlCa, its process is financially costly and invasive. Also, the high recurrence rate of BlCa requires frequent and prolonged surveillance. So, it needs non-invasive and more economical diagnostic methods, which cystoscopy could not meet. Urinary methylation markers analysis, as a non-invasive diagnostic method, meets these clinical needs.

Urinary specimens, in direct contact with tumors, is a concrete and easily attainable source of tumor samples. DNA methylation in urine specimens could be initially discovered as an early event in BlCa using quantitative methylation-specific-PCR (qMSP) [4, 5]. Until now, there are numerous researches concerned about the urinary methylation biomarkers (Table 1). Although their methodologies vary, the characteristic of having high sensitivity and specificity was coherently significant.

**Table 1:** Formerly reported high sensitivity and specificity methylation markers in BlCa

Gene	Sample Type	Method	Sampling Size (BlCa/Healthy Urine)	Sensitivity	Specificity	Reference (Year)
**CDKN2A, ARF, MGMT and GSTP1**	Tumor/Urine	qMSP	74/–	82%	96%	[6] (2006)
**GDF15, TMEFF2 and VIM**	Tumor/Urine	qMSP	51/20	94%	90%	[7] (2010)
**TWIST1 and NID2**	Tumor/Urine	qMSP	65/–	88%, 94%	91%, 94%	[8] (2010)
**ZNF154, HOXA9, POU4F2 and EOMES**	Tumor/Urine	MS-HRM	86/–	84%	96%	[9] (2011)
**TCF21 and PCDH17**	Tumor/Urine	qMSP	50/48	60%	100%	[10] (2011)
**MYO3A, CA10, NKX6-2, DBC1 and SOX11 or PENK**	Urine	qMSP	128/39	85%	95%	[11] (2011)
**DAPK, IRF8, p14, RASSF1A and SFRP1**	Urine	qMSP	30/19	86.7%	94.7%	[12] (2011)
**SOX1, IRAK3 and L1-MET**	Urine	qMSP	90/–	93%/80%	94%/97%	[13] (2014)
**CFTR, VAX1, KCNV1, TAL1 and PPOX1**	Urine	qMSP	212/–	88.68%	90.0%	[14] (2012)
**SOX1, TJP2, MYOD, HOXA9_1, HOXA9_2, VAMP8, CASP8 and SPP1**	Tumor/Urine	PSQ	73/18	100%	100%	[15] (2013)
**OSR1, SIM2, OTX1, MEIS1 and ONECUT2**	Urine	Ms-SnuPE	54/–	85%	87%	[16] (2013)

qMSP: quantitative methylation-specific polymerase chain reaction; HRM: high resolution melt; PSQ: pyrosequencing; Ms-SnuPE: methylation sensitive single nucleotide primer extension.

**Figure 1:**
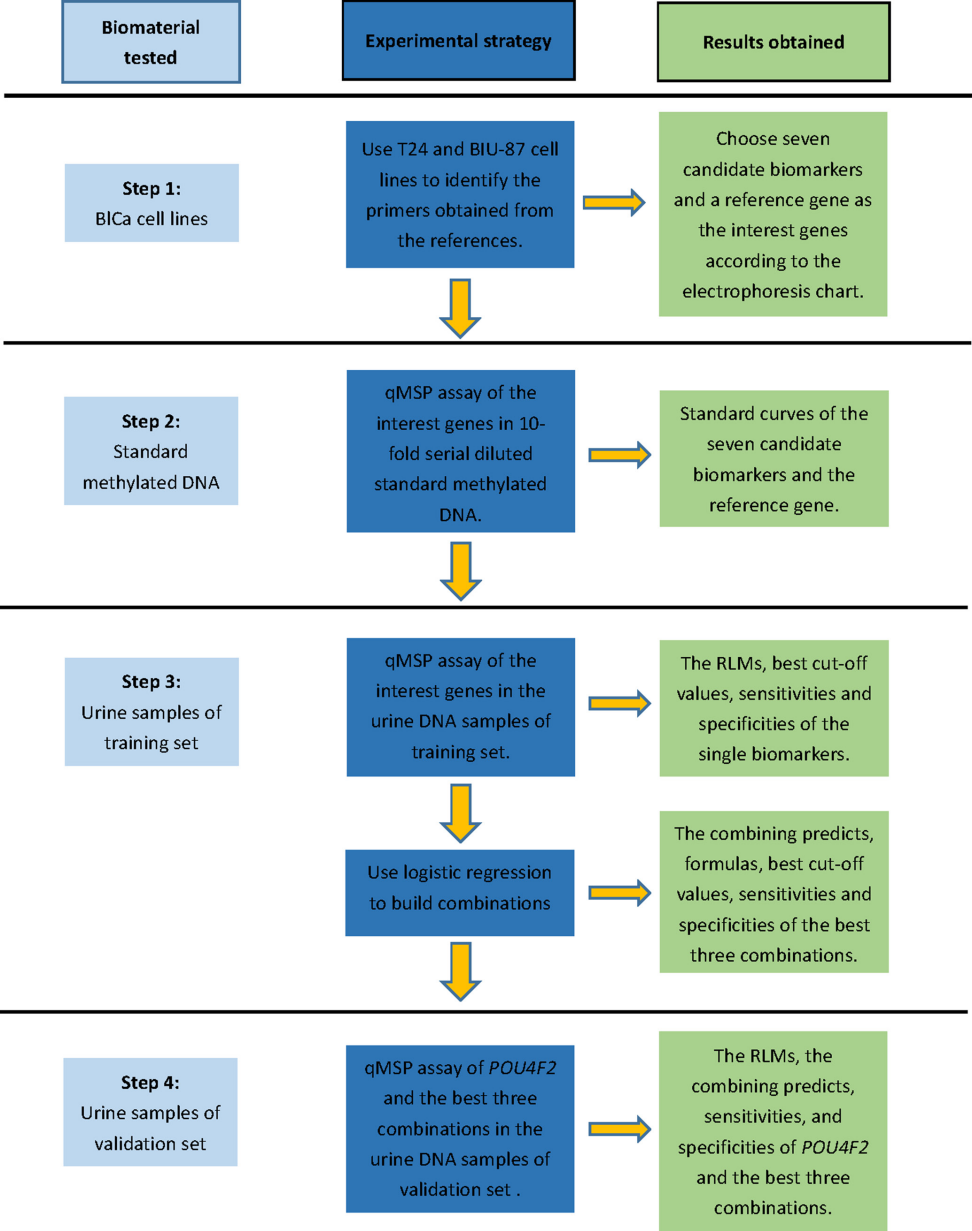
Procedural route map of this study

However, several major uncertainties in the studies on urinary methylation markers still exist. Firstly, while novel urine methylation markers have been found, studies that combined these specific biomarkers to form a novel panel are still limited. Secondly, most of these studies just test these methylation markers in urine samples from several groups (BlCa patients vs healthy volunteers; BlCa patients vs prostate cancer patients; BlCa patients vs kidney patients; primary BlCa patients vs recurrent BlCa patients). None of them have a diversified control group. Thirdly, some of the previous studies used an unreasonable statistical method that will get a high sensitivity and specificity just in their tested sample group, but can hardly repeat in the other study. Finally, the formerly reported biomarkers had their results mostly based on Caucasian population; whether the sensitivity and specificity of the biomarkers can maintain across ethnic groups is yet to be displayed. Here we tested the methylation biomarkers with considerable high sensitivity and specificity from the former studies that provided the primers sequences by qMSP to detect their methylation level in urine samples from Han Chinese individuals. And the procedural route map is displayed in Figure 1. By using multiple biomarkers concurrently, we expect that it will be able to distinguish bladder cancer patients from various control individuals.

## RESULTS

### Result of RLM for each biomarker in training set

**Figure 2:**
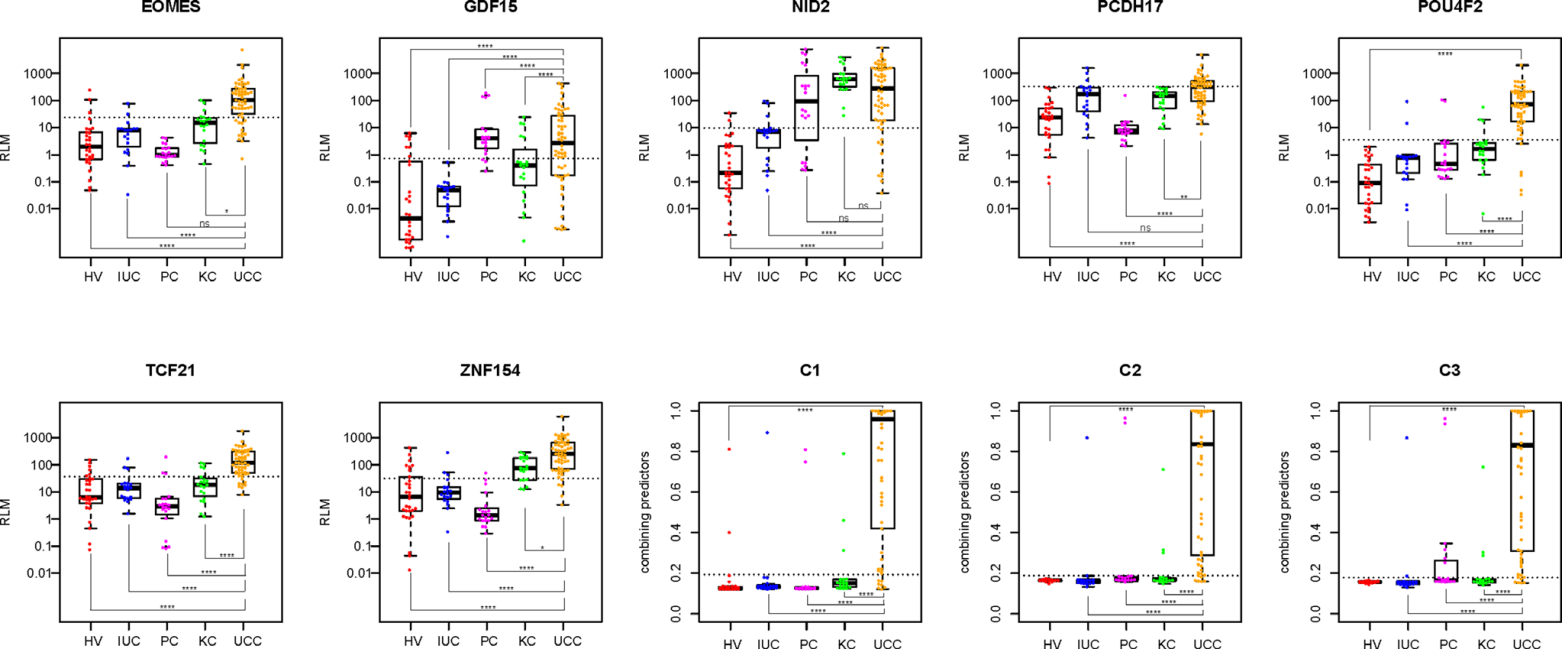
Scatter plots of RLM of interest biomarkers and combining predicts of C1, C2, and C3 in the training set For each scatter plot, the dotted line represents the best cutoff value; Mann-Whitney test was performed across groups: ns = *p* > 0.05, *= *p* ≤ 0.05, **= *p* ≤ 0.01, **** = *p* ≤ 0.0001; HV: healthy volunteer, IUC: infected urinary calculi, PC: prostate cancer; KC: kidney cancer, UCC: urothelial cell carcinoma.

**Figure 3:**
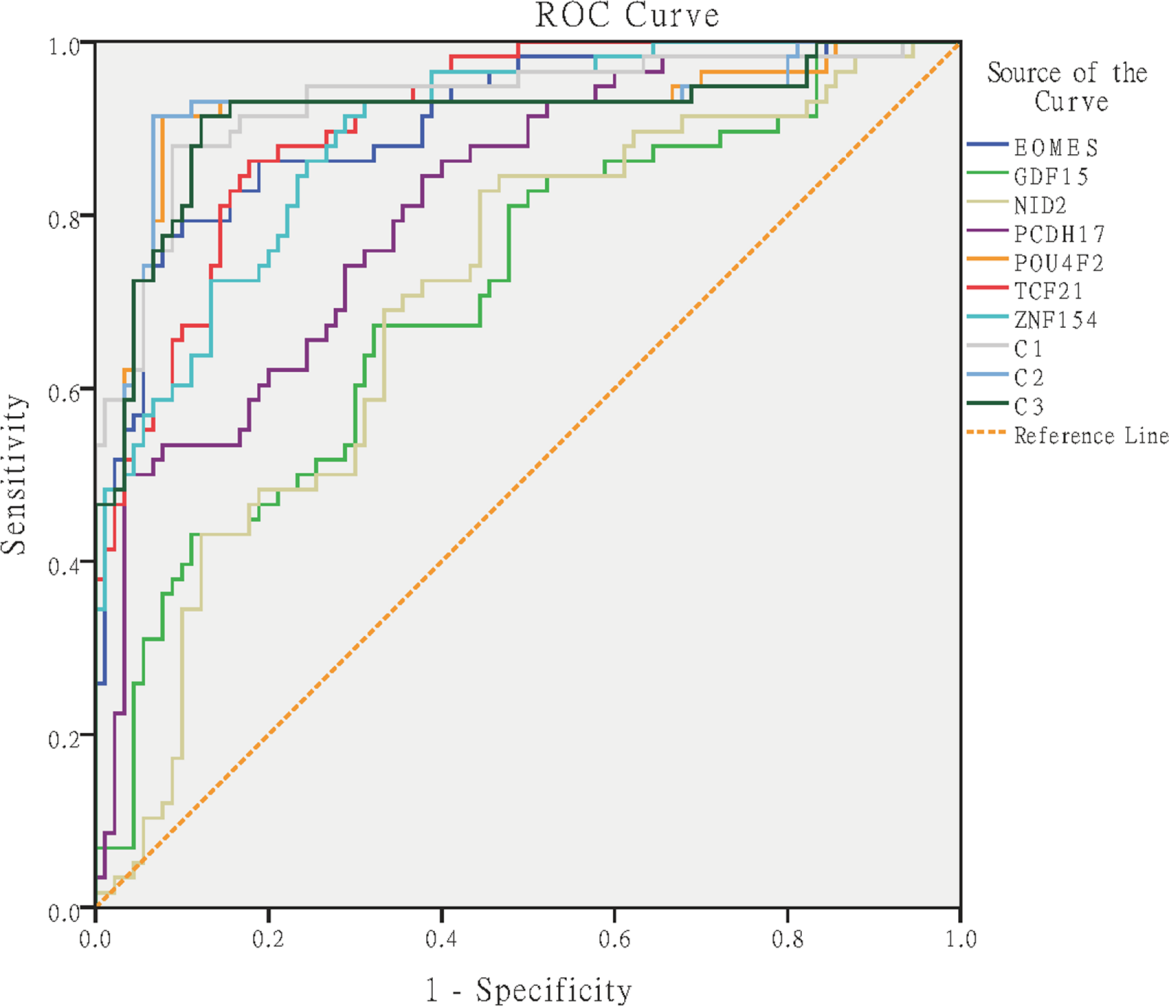
ROC curves for the interest biomarkers and top three combinations C1: POU4F2 + EOMES, C2: POU4F2 + PCDH17, C3: POU4F2 + PCDH17 + GDF15.

The standard curves of the eight genes (*ALU-C4*, *EOMES*, *GDF15*, *NID2*, *PCDH17*, *POU4F2*, *TCF21* and *ZNF154*) were constructed by using the method reported by Lee *et al.* [17]. Each standard curve was linear in the range tested (R^2^ > 0.99) by the duplicate reactions (Supplementary Figure 1). The RLM of each qMSP reaction was estimated with the Ct value of qMSP and the slope of each standard curve. Status of RLM of different sample groups for each biomarker in the training set were displayed (Figure 2). ROC curves of the biomarkers were then constructed (Figure 3). The best cut-off values to discriminate UCC from control groups using each marker were determined from the ROC curves as the maximum values of sensitivity and specificity, as follows: (sensitivity + specificity). The best cut-off value, sensitivity, specificity, area under roc curve (AUC), 95% confidence interval (95% CI), positive predictive value (PPV), and negative predictive value (NPV) of each marker were displayed in Table 2.

**Table 2:** Diagnostic significance of the interest biomarkers and combinations in the training set

Gene	Best cutoff value	Sensitivity, % (pos./total)	Specificity, % (neg./total)	AUC (95% CI)	PPV^a^, % (true pos./total pos.)	NPV^b^, % (true neg./total neg.)
**EOMES**	23.9309	79.31% (46/58)	90.00% (81/90)	0.906 (0.856–0.956)	83.64% (46/55)	87.10% (81/93)
**GDF15**	0.7223	67.24% (39/58)	67.78% (61/90)	0.711 (0.625–0.796)	57.35% (39/68)	76.25% (61/80)
**NID2**	9.7703	82.76% (48/58)	55.56% (50/90)	0.703 (0.617–0.788)	54.55% (48/88)	83.33% (50/60)
**PCDH17**	336.3518	50.00% (29/58)	96.67% (87/90)	0.813 (0.744–0.882)	90.63% (29/32)	75.00% (87/116)
**POU4F2**	3.56	91.38% (53/58)	92.22% (83/90)	0.921 (0.867–0.975)	88.33% (53/60)	94.32% (83/88)
**TCF21**	36.6959	86.21% (50/58)	82.22% (74/90)	0.910 (0.866–0.954)	75.76% (50/66)	90.24% (74/82)
**ZNF154**	31.8368	91.38% (53/58)	71.11% (64/90)	0.892 (0.842–0.942)	67.09% (53/79)	92.75% (64/69)
**C1**	0.1928	87.93% (51/58)	91.11% (82/90)	0.930 (0.884–0.976)	86.44% (51/59)	92.13% (82/89)
**C2**	0.1878	91.38% (53/58)	93.33% (84/90)	0.923(0.869–0.976)	89.83% (53/59)	94.38% (84/89)
**C3**	0.1784	91.38% (53/58)	87.78% (79/90)	0.914 (0.859–0.969)	82.81% (53/64)	94.05% (79/84)

a Positive predictive value.

b Negative predictive value.

### Calculation and selection of the combinations in training set

We used the logistic regression model to combine multiple biomarkers and generate the combining predictors (C) to explore the synergetic potential effect. Among all the possible combinations that can be constituted by the seven biomarkers. The top three combinations with highest sensitivity and specificity were:

$$POU4F2+EOMES\;C1=\frac{1}{1+e^{\left(1.991577-0.014203*EOMES-0.0326B7*POU4F2\right)}}$$

$$POU4F2+PCDH17\;C2=\frac{1}{1+e^{\left(1.624926+0.000502*PCDH17-0.04679*POU4F2\right)}}$$

POU4F2+PCDH17+GDF15

$$C3=\frac{1}{1+e^{\left(1.689007-0.046508*POU4F2-0.000445*PCDH17-0.004995*CDF15\right)}}$$

The performance of combining predictors in the training set for the top three combinations were displayed (Figure 2), and the ROC curves of the combinations were subsequently constructed (Figure 3). The best cut-off values to discriminate UCC from control groups using each combination were determined from the ROC curves as the maximum values of sensitivity and specificity, as follows: (sensitivity + specificity). The best cut-off value, sensitivity, specificity, area under roc curve (AUC), 95% confidence interval (95% CI), positive predictive value (PPV), and negative predictive value (NPV) of each combination were displayed in Table 2.

### *POU4F2* and the top three combinations in the validation set

**Figure 4:**
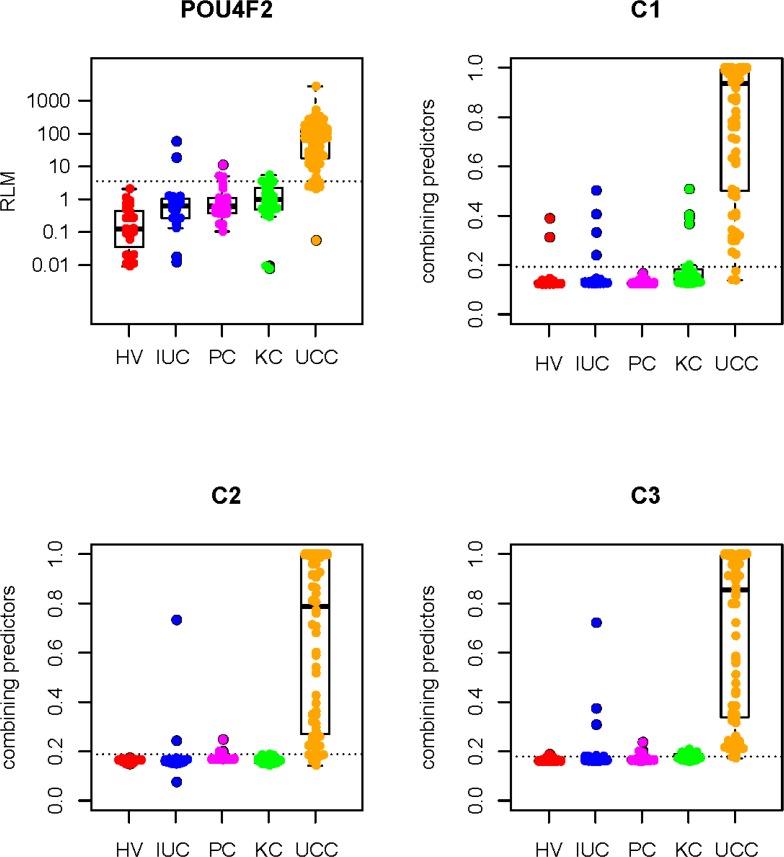
Scatter plots of RLM of POU4F2 and combining predicts of C1, C2, and C3 in the validation set For each scatter plot, the dotted line represents the best cutoff value; Mann-Whitney test was performed between UCC group and HV, IUC, PC, and KC group respectively, all the *P* values are lesser than 0.0001; HV: healthy volunteer, IUC: infected urinary calculi, PC: prostate cancer; KC: kidney cancer, UCC: urothelial cell carcinoma.

In the training set, the single biomarker that has demonstrated the best performance is *POU4F2*, which has the highest sensitivity and specificity of 91.38% and 92.22%. Compared to the single biomarker, the three chosen combinations revealed sensitivity and specificity of 87.93% and 91.11% from *POU4F2* + *EOMES* (C1), 91.38% and 93.33% from *POU4F2* + *PCDH17* (C2), and 93.18% and 87.78% from *POU4F2* + *PCDH17* + *GDF15* (C3) consequently. So, we tested *POU4F2* and the top three combinations in the validation set. The results were displayed in Figure 4. And the sensitivity, specificity, PPV, and NPV were listed in Table 3.

**Table 3:** Diagnostic significance of *POU4F2*, C1, C2, and C3 in the validation set

Gene	Sensitivity, % (pos./total)	Specificity, % (neg./total)	PPV^a^, % (true pos./total pos.)	NPV^b^, % (true neg./total neg.)
**POU4F2**	88.89% (64/72)	93.48% (86/92)	91.43% (64/70)	91.49% (86/94)
**C1**	95.83% (69/72)	88.04% (81/92)	86.25% (69/80)	96.43% (81/84)
**C2**	88.89% (64/72)	94.57% (87/92)	92.75% (64/69)	91.58% (87/95)
**C3**	97.22% (70/72)	75.00% (69/92)	75.27% (70/93)	97.18% (69/71)

a Positive predictive value.

b Negative predictive value.

### *POU4F2* and the top three combinations in all the urine samples (training and validation sets combined)

We combined the training and validation set to find the best marker or combination. The diagnostic significance of *POU4F2* and the top three combinations were listed in Table 4. Of all the urine samples, the best marker or combination is C2 (*POU4F2* + *PCDH17*), with the sensitivity and specificity of 90.00% and 93.96%.

**Table 4:** Diagnostic significance of *POU4F2*, C1, C2, and C3 in the training and validation sets together

Gene	Sensitivity, % (pos./total)	Specificity, % (neg./total)	PPV^a^, % (true pos./total pos.)	NPV^b^, % (true neg./total neg.)
**POU4F2**	90.00% (117/130)	92.86% (169/182)	90.00% (117/130)	92.86% (169/182)
**C1**	92.31% (120/130)	89.56% (163/182)	86.33% (120/139)	94.22% (163/173)
**C2**	90.00% (117/130)	93.96% (171/182)	91.41% (117/128)	92.93% (171/184)
**C3**	94.62% (123/130)	81.32% (148/182)	78.34% (123/157)	95.48% (148/155)

a Positive predictive value.

b Negative predictive value.

### Sensitivity of *POU4F2*, C1, C2 and C3 in each sub-category of bladder cancer patients

In order to determinate whether *POU4F2*, C1, C2 and C3 can be utilized to diagnose bladder cancer in early-stage, we listed their sensitivities in different sub-categories of bladder cancer patients (Table 5). All of them had relatively a high sensitivity (at least 92.50%) in non-muscle invasive bladder cancer (NMIBC) patients.

**Table 5:** Sensitivity of *POU4F2*, C1, C2, and C3 in each sub-category of bladder cancer patients

Sub-category	Sensitivity, % (pos./total)			
	POU4F2	C1	C2	C3
**TNM**				
**Ta-T1**	92.50% (74/80)	92.50% (74/80)	92.50% (74/80)	96.25% (77/80)
**T2-T4**	86.00% (43/50)	92.00% (46/50)	86.00% (43/50)	92.00% (46/50)
**Grade**				
**High**	86.11% (31/36)	91.67% (33/36)	86.11% (31/36)	97.22% (35/36)
**Low**	91.49% (86/94)	92.55% (87/94)	91.49% (86/94)	93.62% (88/94)
**Primary/recurrent**				
**Primary**	90.10% (91/101)	92.08% (93/101)	90.10% (91/101)	94.06% (95/101)
**Recurrent**	89.66% (26/29)	93.10% (27/29)	89.66% (26/29)	96.55% (28/29)

## DISCUSSION

Bladder cancer can be pathologically classified into urothelial cell carcinoma (UCC), squamous cell carcinoma, adenocarcinoma, small cell carcinoma, and other sarcomas. Among them, UCC is the most-common type, which takes 90% of the patient population. Clinically, most of the UCC patients receive diagnostic procedures after certain symptoms have already appeared, such as hematuria. These symptoms usually indicate that the incidence of the tumor has happened for a considerable amount of time. In these cases, conventional radiology examination can fail to distinguish the tumor body due to its lesser size. Assays such as cystoscopy are not appropriate for large-scale inspection for the high-risk population because of their invasive and economically expensive nature. Exfoliative urinary cytology, with its non-invasive nature, still has a poor sensitivity for the diagnosis of early stage bladder cancer. Thus, a novel non-invasive assay that can provide high sensitivity and specificity is extremely necessary.

One of the vital factors that makes non-invasive bladder cancer inspection possible is the unique physiological environment of the bladder. The epithelial cells in the bladder often exfoliate and exit the body through urine. These urinary exfoliative cells can be isolated and then used in examinations. Until now, the non-invasive examinations that have been approved by FDA include exfoliative urinary cytology, bladder tumor antigen (BTA) assay, nuclear matrix protein 22 (NMP22) assay, ImmunoCyt assay, and fluorescent *in situ* hybridization (FISH). These methodologies, due to their strict requirements of sampling and the long period of the process, are not appropriate for the massive and routine application.

The alterations in the state of DNA methylation usually happen more frequently than the mutations in the DNA sequence, especially as a result of changes in the microenvironment. These alterations can be detectable prior to oncogenesis. Thus, assays aiming at them are highly applicable for the inspection on the high-risk population, and can direct prevention measurements prior to the development of the tumor [11]. Numerous previous studies have discussed the methylation biomarkers specific to bladder cancer, and the momentous result of using MSP and qMSP on urine samples to distinguish bladder cancer patients and healthy individuals [7, 8, 10, 11, 18, 19]. However, these studies mainly focused on Caucasian ethnic groups, leaving the methylation status of the corresponding biomarkers in Eastern Asian ethnic groups largely unstudied. Since the methylation status of genes can be inconsistent across different ethnic groups, the native methylation state in Asian populations must be fully analyzed before we can apply the methylation biomarker assay on them.

After following the 4 step strategy (Figure 1), we found that *POU4F2* has the highest reliability when being used individually, resulting a sensitivity of 90.00% and a specificity of 92.86%. In a study carried out by Reinert *et al.*, *POU4F2* also yielded a relatively high sensitivity of 85% and a specificity of 94% [9]. However, it only compared the methylation state of the biomarkers in BlCa patients and healthy volunteers. In this study, we added IUC patients as additional control groups. Samples from these patients helped us to understand whether the high methylation level of the biomarkers we detected was due to the inflammatory cells. We also added KC and PC patients as additional control groups to determinate whether these biomarkers can distinguish UCC from KC and PC.

In addition to utilizing the biomarkers individually, the sensitivity and specificity of numerous combinations of biomarkers were also explored and described by us. The particular biomarkers we have used in these combinations are listed. Among these combinations, C2 (*POU4F2* + *PCDH17*) returned the highest reliability of a sensitivity of 90.00% and a specificity of 93.96%, and has surpassed the result of inspecting *POU4F2* individually.

By adopting qMSP as the assaying method, we have successfully proved that the high sensitive and specificity of the selected biomarkers fulfills the standard of a novel epigenetic detecting method for bladder cancer. However, this study only provides the theoretical basis of this method. To proceed to clinical application, not only we need further investigate the viability on a larger sample size, but complications such as success rate of isolation of DNA from the urine samples need to be overcome first. The traditional method of collecting urine samples was to collect in the early morning. The low success rate due to the lysis of the exfoliated cells caused by body temperature and long *ex situ* exposure time could be one of the reasons that caused the undervaluation of epigenetic detecting for bladder cancer. During the early phase of the experiment, our success rate only reached approximately 25% by collecting urine samples in the early morning, which is close to 30% that were reported by Reinert et al. [9]. Some measurements we applied to improve the collection of the samples have elevated the success rate to 75%. Firstly, we don't collect the morning urine. Secondly, we ask the participants to empty their bladder, and then drink a lot of water quickly and do some exercise. Finally, we collected their urine samples and immediately centrifuged at 800 × g and 4°C for 15 minutes. Samples collected in this way contain more exfoliated cells because the retention time of urine in the bladder was reduced, and the exfoliation was intensified during physical exercise. Still, to make this methodology clinically applicable, a standardized way to preserve the sample and isolate the DNA from the exfoliated cells must be defined and discussed.

## MATERIALS AND METHODS

### Collection and processing of urine samples from patients and volunteers

Urine samples of 58 urothelial cell carcinoma (UCC) patients, 20 infected urinary calculi (IUC) patients, 20 kidney cancer (KC) patients, 20 prostate cancer (PC) patients, and 30 healthy volunteers (HV) were collected as training set from the First Affiliated Hospital of Anhui Medical University, Sun Yat-Sen University Cancer Center, and Zhujiang Hospital of Southern Medical University separately from February 2013 to April 2014 (Supplementary Table 1). And another 72 UCC, 21 IUC, 26 KC and 22 PC, and 23 HV urine samples were collected as the validation set from the First Affiliated Hospital of Anhui Medical University, Sun Yat-Sen University Cancer Center, and Zhujiang Hospital of Southern Medical University separately from February 2015 to October 2015 (Supplementary Table 2). UCC is the experimental group, and IUC, KC, PC, and HV are the control groups. Tumor stage of UCC was assessed according to the modified tumor, node, metastasis (TNM) system suggested by UICC International Union Against Cancer. The World Health Organization 2004 malignancy grading system was used for the evaluation of tumor grade. All of the urine samples were collected during noon and were immediately centrifuged at 800 × g and 4°C for 15 minutes. The supernatants of the centrifuged samples were removed, leaving the sediments for DNA isolation. This research had been carried out in accordance with the ethical standards and according to the Declaration of Helsinki and according to national and international guidelines and has been approved by the review board of the Shenzhen Second People's Hospital. Informed consent has been provided with all the participants.

### DNA isolation and quality control

DNA of the urine sediments and the bladder cancer cell lines were achieved respectively by using TIANamp Micro DNA Kit DP316 (TianGen) and TIANamp Blood/Tissue/Cell DNA Kit DP304 (TianGen) and according to the protocol provided. The concentration of DNA was quantified by Qubit^™^ 2.0 fluorometer (Invitrogen). Molecule size of the DNA of each sample was tested by 1% agarose gel electrophoresis. Finally, only the DNA samples with quantity > 200 ng and size > 5 Kb were used to the following experiments.

### Bisulfite modification

DNA samples and CpGenome^™^ Universal Methylated DNA (Millipore) standard DNA were treated by EZ DNA Methylation-Gold^™^ Kit (Zymo Research). The bisulfite modification reaction was executed by 96-Well GeneAmp PCR System 9700 (Applied Biosystems) with the mixture of 150 μl that contains 130 μl of CT conversion reagent (Zymo Research) and 200 ng of DNA template. The condition of the reaction was configured as 98°C for 10 minutes followed by 64°C for 2.5 hours and then on hold at 4°C. 20 μl of M-Elution Buffer (Zymo Research) was used to purify each DNA sample. The purified DNA was stored at −20°C until use.

### Identifying the primers of the candidate biomarkers

**Figure 5:**
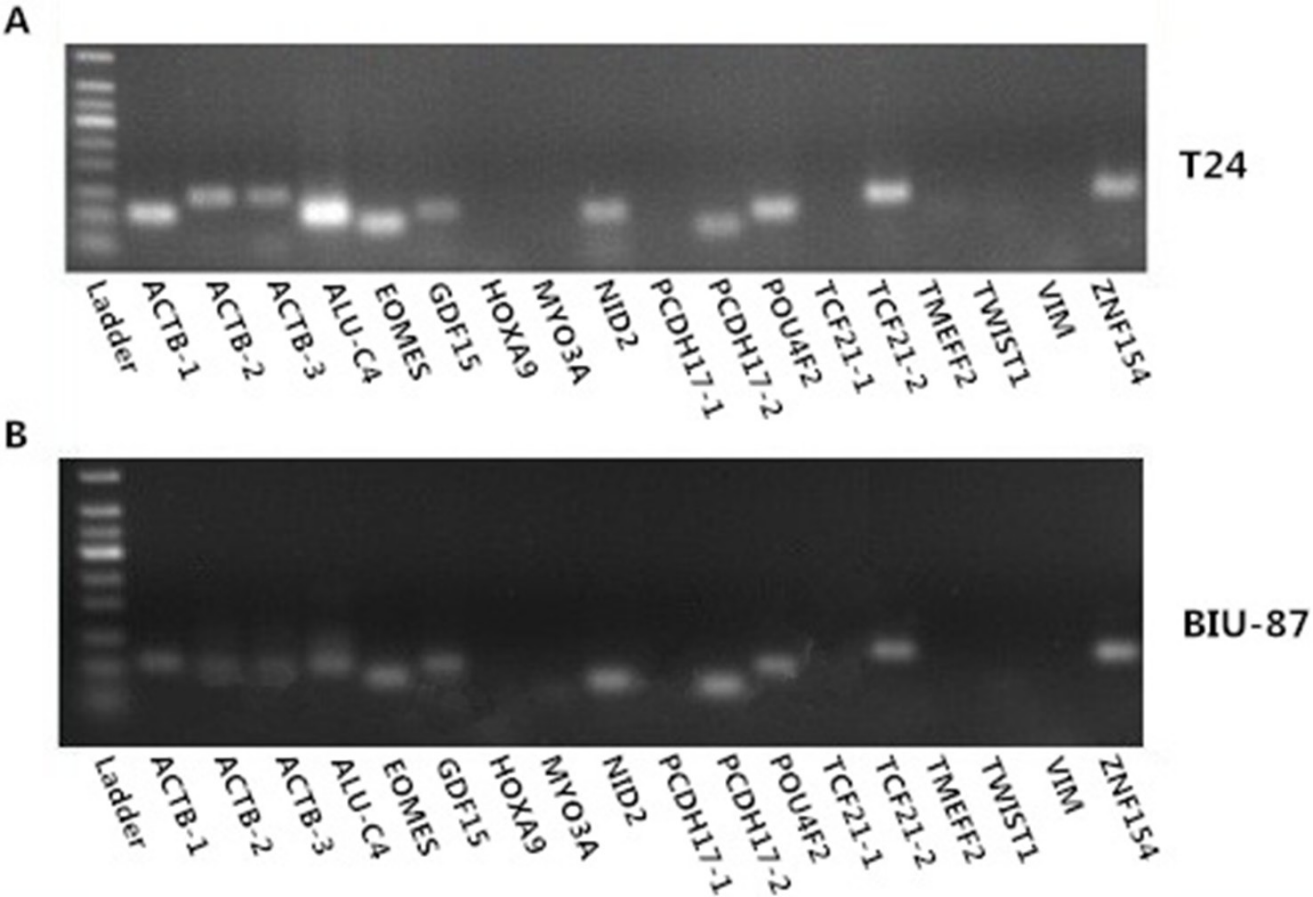
Electrophoresis chart of methylation primers in T24 (A) and BIU-87 (B) bladder cancer cell line DNA samples The ladder is Tiangen 50 bp ladder.

All the 18 pairs of primer (*EOMES*, *GDF15*, *HOXA9*, *MYO3A*, *NID2*, *PCDH17-1*, *PCDH17-2*, *POU4F2*, *TCF21-1*, *TCF21-2*, *TMEFF2*, *TWIST1*, *VIM*, and *ZNF154*; with *ACTB-1*, *ACTB-2*, *ACTB-3*, and *ALU-C4* as reference genes) were used in the PCR (Polymerase Chain Reaction) assay of bisulfite modified T24 and BIU-87 bladder cancer cell line DNA samples (Supplementary Table 3). Each PCR product performed 1.5% agarose gel electrophoresis, and then take photos under the UV imaging system (Figure 5A and 5B). According to the electrophoresis chart, *ACTB-1*, *ACTB-2*, *ACTB-3*, *ALU-C4*, *PCDH17-2*, *POU4F2*, *TCF21-2*, *ZNF154*, *EOMES*, *GDF15*, and *NID2* can be amplified successfully by using bisulfite modified T24 and BIU-87 bladder cancer cell line DNA. So, we choose seven genes (*PCDH17-2*, *POU4F2*, *TCF21-2*, *ZNF154*, *EOMES*, *GDF15*, and *NID2*) as candidate biomarkers. We had not use other bladder cancer cell lines to identify the failed amplified gene primers (*HOXA9*, *MYO3A*, *PCDH17-1*, *TCF21-1*, *TMEFF2*, *TWIST1*, and *VIM*), because most urine DNA samples are not enough for more than 24 qMSP reaction (three times per primer; seven candidate biomarker and a reference gene). We choose *ALU-C4* as the reference gene, because the bands of the *ALU-C4* PCR product are much brighter than *ACTB-1, ACTB-2*, and *ACTB-3*. *PCDH17-2* and *TCF21-2* are named as *PCDH17* and *TCF21* respectively in the following.

### qMSP assay using SYBR green

Primers of seven candidate biomarkers (*PCDH17*, *POU4F2*, *TCF21*, *ZNF154*, *EOMES*, *GDF15* and *NID2*) and *ALU-C4* (reference gene) were used in the qMSP assay of the bisulfite modified DNA samples. Each reaction mixture (20 μl in total) was set to be consisted with 10 μl of SYBR^®^ Premix Ex TaqTM II (TAKARA), 2 μl of 10 μM mixed primers, 0.4 μl of 50 × ROX Reference Dye (TAKARA), 5.6 μl of nuclease-free water, and 2 μl of DNA template. The reactions were performed on StepOnePlus^™^ Real-Time PCR System (Applied Biosystems) after a pre-incubation of 2 minutes at 95°C, and were executed for 45 cycles at 95°C for 15 seconds each, situated at 65°C for 30 seconds, followed by the extension step at 72°C for 30 seconds as one cycle. The fluorescence signal was measured at the end of each extension step at 72°C. Every reaction repeated three times. The mean of the Ct values of three times were used as the final Ct value.

### Construction of standard curves and determination of RLM

Bisulfite modified CpGenome^™^ Universal Methylated DNA (Millipore) were used to perform qMSP, and the PCR products were used to construct the standard curves. The size of each product was identified and selected by agarose gel electrophoresis, and then purified by MinElute Gel Extraction Kit (QIAGEN). The concentration of each purified PCR product was quantified by Qubit^™^ (Invitrogen) and then re-quantified after each was standardized to 1.00 ng/μl. The copy number of the diluted sample was calculated using the following equation [20]:

$$\text{Copy Number=}\frac{6.02\times 10^{23}\text{ copy}\cdot {\text{mol}}^{-1}\times \text{DNA Amount}}{\text{DNA Length}\times 660\text{ g}\cdot {\text{mol}}^{-1}\cdot {\text{bp}}^{-1}}$$

In order to estimate the copy number of each sample, a 10-fold serial dilution of the diluted DNA, ranging from 10 to 1 × 10^7^ copies per μl was used to construct the standard curve for each primer. The reaction system was consisted with 10 μl of SYBR^®^ Premix Ex TaqTM II (TAKARA), 1 μl of 10 μM mixed primers, 0.4 μl of 50 × ROX Reference Dye (TAKARA), 1 μl of DNA template, and 6.6 μl of nuclease-free water. PCR amplification was performed with StepOnePlus^™^ Real-Time PCR System (Applied Biosystems) according to the same program with the qMSP assays. The results of Ct value of qMSP and the copy concentration were used for standard curves construction of each target gene. The exact copy concentration of the target gene was determined by relating the Ct value to the standard curve. The linear formulae of the eight genes of interest can be shown as: C_t_ = *A* × log_10_ (*copy*_#_ + *B*) with A and B as constants. The copy number of the methylated sequences can be calculated by: Copy#=10^[Ct−BA]

The relative level of methylation (RLM) for each interest genes in each sample based on its copy number was calculated on the basis of the method used by Costa *et al.*, which utilizes the formula [7]:

$$\text{RLM}=\frac{{\left(\frac{\text{Copy Number gene}}{\text{Copy Number }ALU}\right)}_{\text{sample}}}{{\left(\frac{\text{Copy Number gene}}{\text{Copy Number }ALU}\right)}_{\text{standard}}}\times 100$$

After simplification, the final formula for RLM calculation is:

$$\text{RLM}=10\left(\frac{{\text{Ct}}_{\text{gene-sample}}-{\text{Ct}}_{\text{gene-standard}}}{A_{\text{gene}}}+\frac{{\text{Ct}}_{\text{ALU-standard}}-{\text{Ct}}_{\text{ALU-sample}}}{A_{\text{ALU}}}\right)\times 100$$

The RLM was used as a final result of qMSP for the data statistical procedures.

### Statistical analysis

The resulted RLM were analyzed by SPSS 21.0 software (IBM). The significance of differences in RLM in diverse groups was assessed by pairwise comparisons using the Mann-Whitney test. ROC curves were built for biomarkers and combinations by plotting the true positive rate (sensitivity) against the false positive rate (1 – specificity). The area under the curve (AUC) value and the 95% confidence interval (95% CI) were also calculated. If the 2-tailed *p*-value derived from the statistical test was less than 0.05, then the test is considered to be statistically significant.

## SUPPLEMENTARY MATERIAL FIGURE AND TABLES


