# Supplementary material for: An epigenetic biomarker combination of PCDH17 and POU4F2 detects bladder cancer accurately by methylation analyses of urine sediment DNA in Han Chinese
Source: Oncotarget. 2015 Dec 19;7(3):2754–64. doi: 10.18632/oncotarget.6666 (PMC4823069; doi:10.18632/oncotarget.6666)
Supplement: Supplementary file 1 [file oncotarget-07-2754-s001.pdf]

An epigenetic biomarker combination of PCDH17 and POU4F2 detects bladder cancer accurately by methylation analyses of urine sediment DNA in Han Chinese

Supplementary Materials

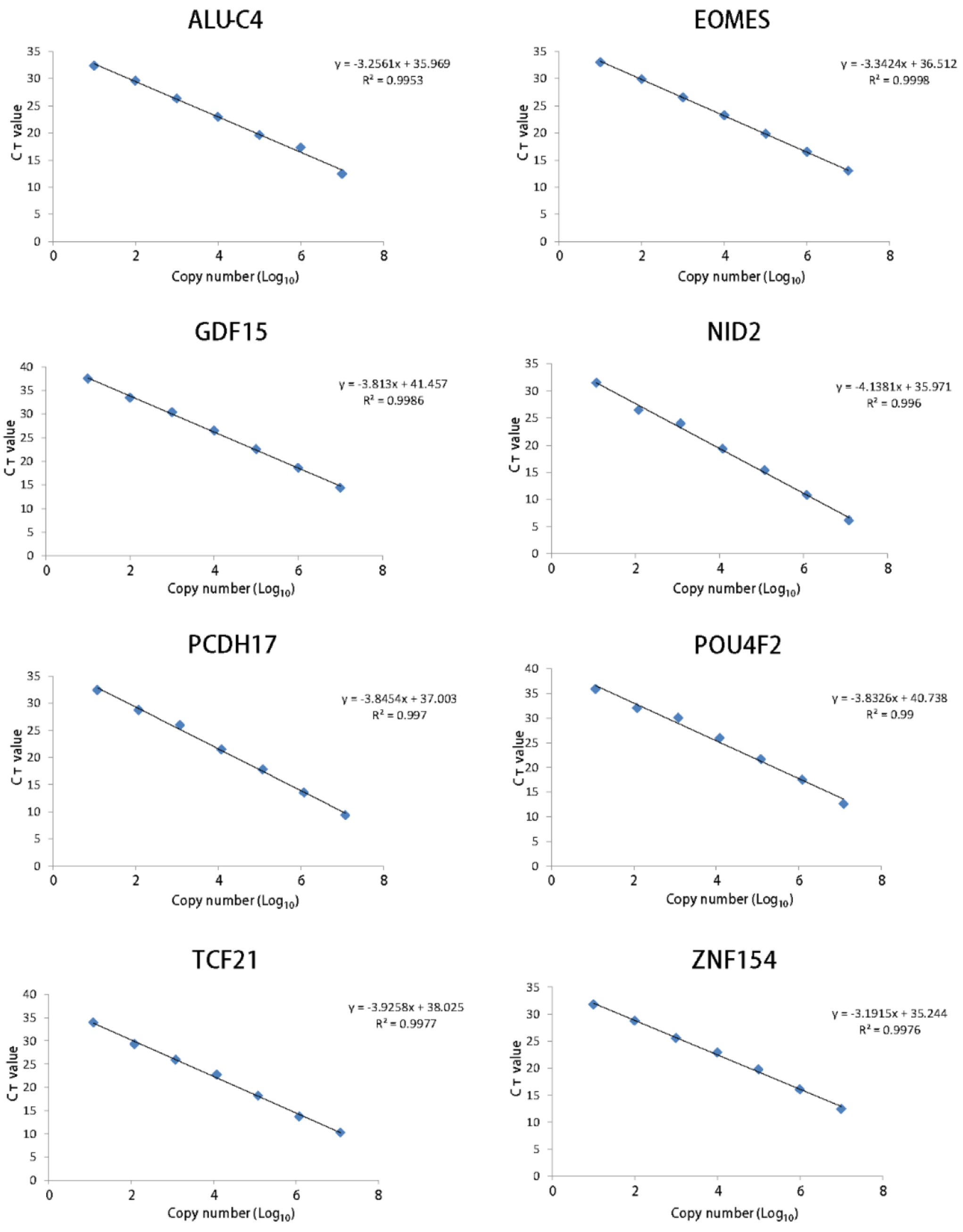

Supplementary Figure S1: Standard curves of the candidate genes.

Supplementary Table S1: Clinical information of the training set

Case ID	Gender	Age	Grade	TNM	Primary/ recurrent
**UCC01**	male	64	high	T4N1M0	primary
**UCC02**	male	73	high	T2bN0M0	primary
**UCC03**	female	63	high	TaN0M0	primary
**UCC04**	female	40	high	T2bN0M0	primary
**UCC05**	male	40	low	T1N0M0	primary
**UCC06**	female	46	high	TaN0M0	primary
**UCC07**	female	62	low	T1N0M0	recurrent
**UCC08**	female	33	high	T1N0M0	primary
**UCC09**	female	72	high	T3bN0M0	primary
**UCC10**	male	73	high	TaN0M0	primary
**UCC11**	male	70	high	T1N0M0	primary
**UCC12**	male	61	high	T2N0M0	primary
**UCC13**	male	75	high	T2bN0M0	primary
**UCC14**	male	50	low	T1N0M0	recurrent
**UCC15**	female	73	high	T2bN0M0	primary
**UCC16**	male	50	low	T2aN0M0	primary
**UCC17**	male	56	high	T1N0M0	recurrent
**UCC18**	male	43	low	TaN0M0	primary
**UCC19**	male	59	high	T3aN0M0	primary
**UCC20**	female	55	low	TaN0M0	recurrent
**UCC21**	male	58	high	T2aN0M0	primary
**UCC22**	male	35	low	T1N0M0	recurrent
**UCC23**	male	66	high	T1N0M0	recurrent
**UCC24**	male	59	high	T1N0M0	primary
**UCC25**	male	74	high	TaN0M0	primary
**UCC26**	male	49	high	T2aN0M0	recurrent
**UCC27**	female	41	high	T3bN3M1	primary
**UCC28**	male	68	high	TaN0M0	recurrent
**UCC29**	male	74	high	T2aN0M0	primary
**UCC30**	male	61	high	T1N0M0	primary
**UCC31**	male	74	high	T4aN2M0	primary
**UCC32**	male	54	high	T1N0M0	primary
**UCC33**	male	71	low	TaN0M0	primary
**UCC34**	male	70	high	T1N0M0	recurrent
**UCC35**	male	68	high	T2N0M0	recurrent
**UCC36**	male	87	high	T1N0M0	primary
**UCC37**	male	55	high	T1N0M0	primary

**UCC38**	male	66	high	T1N0M0	primary
**UCC39**	male	48	low	T1N0M0	primary
**UCC40**	male	54	low	TaN0M0	primary
**UCC41**	female	75	high	T1N0M0	recurrent
**UCC42**	male	79	low	TaN0M0	primary
**UCC43**	male	67	high	T2N0M0	primary
**UCC44**	female	80	low	TaN0M0	primary
**UCC45**	male	74	high	T2bN0M0	primary
**UCC46**	male	45	high	T3N0M0	primary
**UCC47**	female	41	low	T1N0M0	primary
**UCC48**	male	61	high	T1N0M0	primary
**UCC49**	male	74	high	T4aN2M0	primary
**UCC50**	male	49	low	TaN0M0	primary
**UCC51**	male	60	high	T2bN0M0	primary
**UCC52**	male	66	high	T3aN1M0	recurrent
**UCC53**	female	36	high	T3aN0M0	primary
**UCC54**	female	63	low	TaN0M0	primary
**UCC55**	female	72	low	T1N0M0	primary
**UCC56**	male	83	low	T1N0M0	primary
**UCC57**	male	61	high	T2bN0M0	primary
**UCC58**	male	35	high	T1N0M0	recurrent
**HV01**	female	25	NA	NA	NA
**HV02**	female	27	NA	NA	NA
**HV03**	male	27	NA	NA	NA
**HV04**	male	28	NA	NA	NA
**HV05**	female	28	NA	NA	NA
**HV06**	male	28	NA	NA	NA
**HV07**	male	27	NA	NA	NA
**HV08**	female	29	NA	NA	NA
**HV09**	female	29	NA	NA	NA
**HV10**	male	26	NA	NA	NA
**HV11**	female	30	NA	NA	NA
**HV12**	male	26	NA	NA	NA
**HV13**	male	28	NA	NA	NA
**HV14**	male	28	NA	NA	NA
**HV15**	male	28	NA	NA	NA
**HV15**	male	28	NA	NA	NA
**HV16**	male	31	NA	NA	NA
**HV17**	male	28	NA	NA	NA

**HV18**	male	26	NA	NA	NA
**HV19**	male	30	NA	NA	NA
**HV20**	male	28	NA	NA	NA
**HV21**	male	28	NA	NA	NA
**HV22**	male	29	NA	NA	NA
**HV23**	male	26	NA	NA	NA
**HV24**	male	28	NA	NA	NA
**HV26**	male	26	NA	NA	NA
**HV27**	male	30	NA	NA	NA
**HV28**	male	27	NA	NA	NA
**HV29**	male	30	NA	NA	NA
**HV30**	male	30	NA	NA	NA
**IUC01**	female	41	NA	NA	NA
**IUC02**	female	61	NA	NA	NA
**IUC03**	male	55	NA	NA	NA
**IUC04**	male	46	NA	NA	NA
**IUC05**	female	24	NA	NA	NA
**IUC06**	male	46	NA	NA	NA
**IUC07**	female	30	NA	NA	NA
**IUC08**	female	85	NA	NA	NA
**IUC09**	male	30	NA	NA	NA
**IUC10**	female	60	NA	NA	NA
**IUC11**	female	25	NA	NA	NA
**IUC12**	female	34	NA	NA	NA
**IUC13**	female	53	NA	NA	NA
**IUC14**	female	24	NA	NA	NA
**IUC15**	female	40	NA	NA	NA
**IUC16**	male	51	NA	NA	NA
**IUC17**	male	85	NA	NA	NA
**IUC18**	female	78	NA	NA	NA
**IUC19**	male	83	NA	NA	NA
**IUC20**	female	44	NA	NA	NA
**KC01**	female	79	NA	NA	NA
**KC02**	male	43	NA	NA	NA
**KC03**	male	56	NA	NA	NA
**KC04**	male	38	NA	NA	NA
**KC05**	female	71	NA	NA	NA
**KC06**	male	62	NA	NA	NA
**KC07**	female	38	NA	NA	NA

**KC08**	female	78	NA	NA	NA
**KC09**	male	82	NA	NA	NA
**KC10**	male	31	NA	NA	NA
**KC11**	male	62	NA	NA	NA
**KC12**	female	48	NA	NA	NA
**KC13**	female	59	NA	NA	NA
**KC14**	female	28	NA	NA	NA
**KC15**	female	57	NA	NA	NA
**KC16**	female	38	NA	NA	NA
**KC17**	male	62	NA	NA	NA
**KC18**	female	66	NA	NA	NA
**KC19**	female	50	NA	NA	NA
**KC20**	male	22	NA	NA	NA
**PC01**	male	72	NA	NA	NA
**PC02**	male	83	NA	NA	NA
**PC03**	male	72	NA	NA	NA
**PC04**	male	75	NA	NA	NA
**PC05**	male	68	NA	NA	NA
**PC06**	male	73	NA	NA	NA
**PC07**	male	70	NA	NA	NA
**PC08**	male	70	NA	NA	NA
**PC09**	male	68	NA	NA	NA
**PC10**	male	66	NA	NA	NA
**PC11**	male	73	NA	NA	NA
**PC12**	male	72	NA	NA	NA
**PC13**	male	74	NA	NA	NA
**PC14**	male	68	NA	NA	NA
**PC15**	male	70	NA	NA	NA
**PC16**	male	78	NA	NA	NA
**PC17**	male	67	NA	NA	NA
**PC18**	male	70	NA	NA	NA
**PC19**	male	65	NA	NA	NA
**PC20**	male	70	NA	NA	NA

UCC: Urothelial cell carcinoma; HV: Healthy volunteers; IUC: Infected urinary calculi; KC: Kidney cancer; PC: Prostate cancer.

Supplementary Table S2: Clinical information of the validation set

Case ID	gender	age	grade	TNM	primary/ recurrent
**UCC01**	male	43	high	T2bN0M0	primary
**UCC02**	male	75	high	T2bN0M0	primary
**UCC03**	female	56	high	T2aN0M0	recurrent
**UCC04**	male	43	low	TaN0M0	primary
**UCC05**	female	65	high	T2N0M0	recurrent
**UCC06**	male	62	high	TaN0M0	primary
**UCC07**	male	56	low	TaN0M0	primary
**UCC08**	male	70	high	T1N0M0	primary
**UCC09**	male	64	high	T1N0M0	recurrent
**UCC10**	male	49	high	T2aN0M0	recurrent
**UCC11**	male	72	high	T3bN2M0	primary
**UCC12**	male	57	high	T3aN0M0	primary
**UCC13**	male	51	high	T3aN0M0	primary
**UCC14**	male	57	high	T1N0M0	primary
**UCC15**	male	59	high	T1N0M0	primary
**UCC16**	female	81	high	T1N0M0	primary
**UCC17**	male	50	high	T2N0M0	recurrent
**UCC18**	male	59	high	T3N0M0	primary
**UCC19**	male	80	high	T1N0M0	primary
**UCC20**	male	55	high	T3bN0M0	primary
**UCC21**	female	55	low	T1N0M0	primary
**UCC22**	male	61	high	T2bN0M0	primary
**UCC23**	male	73	high	T1N0M0	primary
**UCC24**	male	57	low	TaN0M0	recurrent
**UCC25**	male	39	low	T2aN0M0	primary
**UCC26**	male	67	high	T1N0M0	primary
**UCC27**	female	62	low	TaN0M0	recurrent
**UCC28**	female	65	low	TaN0M0	primary
**UCC29**	male	70	high	T1N0M0	primary
**UCC30**	male	58	low	T1N0M0	primary
**UCC31**	male	80	high	T1N0M0	primary
**UCC32**	male	70	high	T1N0M0	primary
**UCC33**	male	65	high	T1N0M0	primary
**UCC34**	male	69	high	TaN0M0	primary
**UCC35**	female	53	low	T1N0M0	primary
**UCC36**	female	63	high	TaN0M0	primary
**UCC37**	male	36	high	T2aN0M0	primary
**UCC38**	male	71	high	TaN0M0	primary

**UCC39**	male	59	low	T2aN0M0	primary
**UCC40**	male	44	low	TaN0M0	recurrent
**UCC41**	male	70	high	T1N0M0	primary
**UCC42**	female	55	high	T4aN2M0	primary
**UCC43**	male	47	low	TaN0M0	primary
**UCC44**	male	59	high	T1N0M0	recurrent
**UCC45**	male	56	low	T1N0M0	primary
**UCC46**	male	58	high	T2N0M0	recurrent
**UCC47**	male	68	high	T4aN2M0	primary
**UCC48**	male	63	high	T1N0M0	primary
**UCC49**	male	79	high	T2bN0M0	primary
**UCC50**	female	63	low	TaN0M0	primary
**UCC51**	male	58	high	T1N0M0	recurrent
**UCC52**	male	84	high	T1N0M0	recurrent
**UCC53**	male	60	high	T3aN1M0	recurrent
**UCC54**	male	62	high	T3aN1M0	recurrent
**UCC55**	male	63	high	T3aN1M0	recurrent
**UCC56**	female	63	high	T2bN0M0	primary
**UCC57**	female	65	low	T1N0M0	primary
**UCC58**	male	63	high	T1N0M0	primary
**UCC59**	male	53	low	TaN0M0	primary
**UCC60**	male	60	high	T1N0M0	primary
**UCC61**	male	74	high	T1N0M0	primary
**UCC62**	male	67	high	TaN0M0	primary
**UCC63**	female	55	high	T1N0M0	recurrent
**UCC64**	male	59	high	T4N1M0	primary
**UCC65**	male	72	high	T2N0M0	primary
**UCC66**	male	58	low	TaN0M0	primary
**UCC67**	male	65	low	TaN0M0	primary
**UCC68**	male	70	low	TaN0M0	primary
**UCC69**	male	57	high	T1N0M0	primary
**UCC70**	male	64	high	T2bN0M0	primary
**UCC71**	female	52	high	T4aN2M0	primary
**UCC72**	male	61	high	T1N0M0	primary
**HV01**	female	42	NA	NA	NA
**HV02**	male	31	NA	NA	NA
**HV03**	male	37	NA	NA	NA
**HV04**	female	35	NA	NA	NA
**HV05**	male	34	NA	NA	NA

**HV06**	male	30	NA	NA	NA
**HV07**	male	45	NA	NA	NA
**HV08**	male	28	NA	NA	NA
**HV09**	male	29	NA	NA	NA
**HV10**	male	32	NA	NA	NA
**HV11**	male	43	NA	NA	NA
**HV12**	female	30	NA	NA	NA
**HV13**	female	30	NA	NA	NA
**HV14**	male	33	NA	NA	NA
**HV15**	male	39	NA	NA	NA
**HV16**	female	29	NA	NA	NA
**HV17**	male	44	NA	NA	NA
**HV18**	female	28	NA	NA	NA
**HV19**	female	29	NA	NA	NA
**HV20**	male	29	NA	NA	NA
**HV21**	male	36	NA	NA	NA
**HV22**	male	32	NA	NA	NA
**HV23**	male	35	NA	NA	NA
**IUC01**	female	41	NA	NA	NA
**IUC02**	male	44	NA	NA	NA
**IUC03**	female	59	NA	NA	NA
**IUC04**	female	27	NA	NA	NA
**IUC05**	female	43	NA	NA	NA
**IUC06**	female	38	NA	NA	NA
**IUC07**	male	38	NA	NA	NA
**IUC08**	male	56	NA	NA	NA
**IUC09**	male	58	NA	NA	NA
**IUC10**	female	39	NA	NA	NA
**IUC11**	female	60	NA	NA	NA
**IUC12**	male	51	NA	NA	NA
**IUC13**	male	26	NA	NA	NA
**IUC14**	female	32	NA	NA	NA
**IUC15**	male	41	NA	NA	NA
**IUC16**	male	56	NA	NA	NA
**IUC17**	male	36	NA	NA	NA
**IUC18**	female	60	NA	NA	NA
**IUC19**	female	65	NA	NA	NA
**IUC20**	male	31	NA	NA	NA
**IUC21**	female	33	NA	NA	NA

**KC01**	male	58	NA	NA	NA
**KC02**	male	66	NA	NA	NA
**KC03**	male	54	NA	NA	NA
**KC04**	male	64	NA	NA	NA
**KC05**	male	59	NA	NA	NA
**KC06**	female	67	NA	NA	NA
**KC07**	female	57	NA	NA	NA
**KC08**	female	60	NA	NA	NA
**KC09**	male	52	NA	NA	NA
**KC10**	male	44	NA	NA	NA
**KC11**	female	54	NA	NA	NA
**KC12**	female	62	NA	NA	NA
**KC13**	male	71	NA	NA	NA
**KC14**	female	60	NA	NA	NA
**KC15**	male	66	NA	NA	NA
**KC16**	female	78	NA	NA	NA
**KC17**	male	61	NA	NA	NA
**KC18**	male	52	NA	NA	NA
**KC19**	male	68	NA	NA	NA
**KC20**	female	66	NA	NA	NA
**KC21**	female	67	NA	NA	NA
**KC22**	male	41	NA	NA	NA
**KC23**	female	63	NA	NA	NA
**KC24**	male	65	NA	NA	NA
**KC25**	male	65	NA	NA	NA
**KC26**	male	64	NA	NA	NA
**PC01**	male	70	NA	NA	NA
**PC02**	male	84	NA	NA	NA
**PC03**	male	71	NA	NA	NA
**PC04**	male	70	NA	NA	NA
**PC05**	male	75	NA	NA	NA
**PC06**	male	81	NA	NA	NA
**PC07**	male	81	NA	NA	NA
**PC08**	male	83	NA	NA	NA
**PC09**	male	82	NA	NA	NA
**PC10**	male	82	NA	NA	NA
**PC11**	male	83	NA	NA	NA
**PC12**	male	81	NA	NA	NA
**PC13**	male	69	NA	NA	NA

**PC14**	male	75	NA	NA	NA
**PC15**	male	80	NA	NA	NA
**PC16**	male	75	NA	NA	NA
**PC17**	male	66	NA	NA	NA
**PC18**	male	84	NA	NA	NA
**PC19**	male	68	NA	NA	NA
**PC20**	male	72	NA	NA	NA
**PC21**	male	77	NA	NA	NA
**PC22**	male	69	NA	NA	NA

UCC: Urothelial cell carcinoma; HV: Healthy volunteers; IUC: Infected urinary calculi; KC: Kidney cancer; PC: Prostate cancer.

Supplementary Table S3: List of the primers of methylation biomarkers

Gene	Primers	Product size (bp)
ACTB-1 (Reference gene)	F:TAGGGAGTATATAGGTTGGGGAAGTT<br>R:AACACACAATAACAAACACAAATTCAC	103
ACTB-2 (Reference gene)	F:TGGTGATGGAGGAGGTTTAGTAAGT<br>R:AACCAATAAAACCTACTCCTCCCTTAA	133
ACTB-3 (Reference gene)	F:TGGTGATGGAGGAGGTTTAGTAGGT<br>R:ACCCAATAAAACCTACTCCTCCCTTAA	133
ALU-C4 (Reference gene)	F:GGTTAGGTATAGTGGTTTATATTTGTAATTTTAGTA<br>R:ATTAACTAAACTAATCTTAAACTCCTAACCTCA	89
EOMES	F:GGTTGGGGAAGTAGAGTTTCGAT<br>R:ATAAACAATTACAAACGCCGCCA	80
GDF15	F:TCGGCGGTTATTTGTATTTGC<br>R:CGTCGAAAACAACCGAAACA	101
HOXA9	F:GTGGTTATTATCGTGTTTAGCGT<br>R:CCGATACCACCAAATTATTACATA	110
MYO3A	F:TCGGCGGGAGGATTTGAT<br>R:CCCGCGAACCGAAATAAAA	93
NID2	F:GCGGTTTTTAAGGAGTTTTATTTTC<br>R:CTACGAAATTCCCTTTACGCT	99
PCDH17–1	F:ATAGGTTTGGGATGTA<br>R:GAAAAACAACTAACACTTA	329
PCDH17–2	F:CGGCGGAGGGCGTAGTA<br>R:GTCCACGTCCAACAAATACGATAC	73
POU4F2	F:GTTGTGCGAAGTTGAGTTTATTC<br>R:CCGTTCAAACTAACAACAAAAACGA	142
TCF21–1	F:AGGATTTTTAAGAGGTGG<br>R:AAACCTTACTCAACACTC	267
TCF21–2	F:GTTAAGAGGAGGAAGGCGTTTATT<br>R:CCTTACTCAACACTCGCATACGA	124
TMEFF2	F:GTTCGGGGTTACGCGC<br>R:TTCGCCTCACTCTCCGCT	83

TWIST1	F:GTTAGGGTTCGGGGGCGTTGTT<br>R:CCGTCGCCTTCCTCCGACGAA	92
VIM	F:TTCGGGAGTTAGTTCGCGTT<br>R:ACCGCCGAACATCCTACGA	103
ZNF154	F:TTTATCGGATTAGAGATAGTAGAGCGT<br>R:TAACGTAAATCCCCCAAAACGACG	152

F: forward primer; R: reverse primer.
